# Supplementary material for: Virological failure and risk factors among people living with HIV taking second-line ART in Addis Ababa, Ethiopia
Source: PLoS One. 2026 Feb 2;21(2):e0330581. doi: 10.1371/journal.pone.0330581 (PMC12863470; doi:10.1371/journal.pone.0330581)
Supplement: S2 Fig — (DOCX) [file pone.0330581.s002.docx]

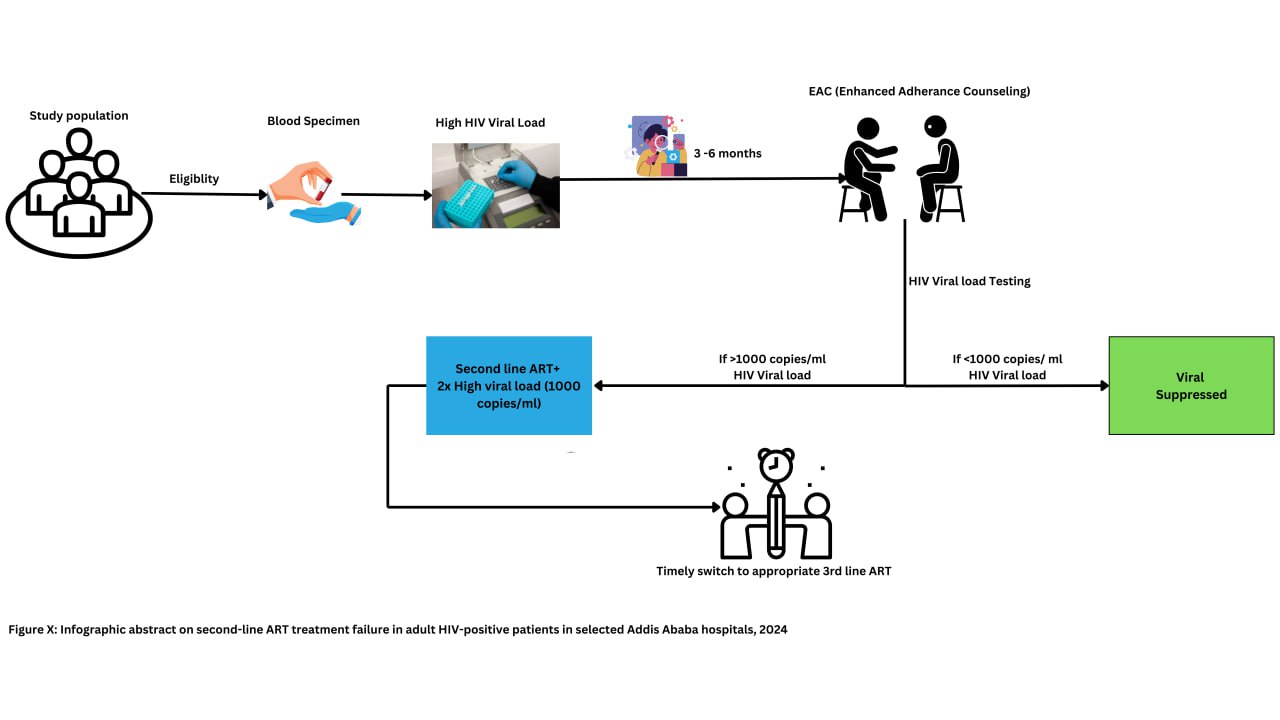


## S2 Fig. Infographic depicting the overall summary of defining virological failure in the research process.
