# Supplementary material for: Virological failure and risk factors among people living with HIV taking second-line ART in Addis Ababa, Ethiopia
Source: PLoS One. 2026 Feb 2;21(2):e0330581. doi: 10.1371/journal.pone.0330581 (PMC12863470; doi:10.1371/journal.pone.0330581)
Supplement: S3 Fig — (DOCX) [file pone.0330581.s003.docx]

S3 Fig. a time range by which the patients developed OI while on second-line ART in Addis Ababa, Ethiopia
