# Supplementary material for: Virological failure and risk factors among people living with HIV taking second-line ART in Addis Ababa, Ethiopia
Source: PLoS One. 2026 Feb 2;21(2):e0330581. doi: 10.1371/journal.pone.0330581 (PMC12863470; doi:10.1371/journal.pone.0330581)
Supplement: Attachment — Submitted filename: Rebutal BB Feyissa (Response to the Reveiwers).R.docx [file pone.0330581.s005.docx]

**Intro:**

*We would like to sincerely thank the editor and the reviewers for the time taken to review our work and provide constructive feedback. Your comments have been instrumental in improving the quality of the manuscript. Below, we detail how we have addressed each point as raised by the editor and reviewers*.

**Response to Journal Requirements.**

1. Please ensure that your manuscript meets PLOS ONE's style requirements, including those for file naming. The PLOS ONE style templates can be found at

**Response:**

*We have noted and ensured our revised manuscript complies with the PLOS ONE style template*

2. Please note that funding information should not appear in any section or other areas of your manuscript.

**Response:**

*We have noted and removed funding information from the revised manuscript*

3. We note that the grant information you provided in the ‘Funding Information’ and ‘Financial Disclosure’ sections do not match. When you resubmit, please ensure that you provide the correct grant numbers for the awards you received for your study in the ‘Funding Information’ section.

**Response:**

*Thank you, revised accordingly.*

4. We note that Figure 2 in your submission contain map/satellite images which may be copyrighted. All PLOS content is published under the Creative Commons Attribution License (CC BY 4.0), which means that the manuscript, images, and Supporting Information files will be freely available online, and any third party is permitted to access, download, copy, distribute, and use these materials in any way, even commercially, with proper attribution. For these reasons, we cannot publish previously copyrighted maps or satellite images created using proprietary data, such as Google software (Google Maps, Street View, and Earth). For more information, see our copyright guidelines: <http://journals.plos.org/plosone/s/licenses-and-copyright>.

 We require you to either (1) present written permission from the copyright holder to publish these figures specifically under the CC BY 4.0 license, or (2) remove the figures from your submission:

 1. You may seek permission from the original copyright holder of Figure 2 to publish the content specifically under the CC BY 4.0 license

 2. If you are unable to obtain permission from the original copyright holder to publish these figures under the CC BY 4.0 license or if the copyright holder’s requirements are incompatible with the CC BY 4.0 license, please either i) remove the figure or ii) supply a replacement figure that complies with the CC BY 4.0 license. Please check copyright information on all replacement figures and update the figure caption with source information. If applicable, please specify in the figure caption text when a figure is similar but not identical to the original image and is therefore for illustrative purposes only.

**Response:**

*Thanks for raising the copyright issue* ***once again*** *regarding Figure 1 (in PONE-D-25-41462R1 version) of the manuscript. Although the figure was not adopted from an online map/ satellite image. Rather, the map was originally constructed using the ArcMap 10.4.1 software from the available shape files of Ethiopia. Just for simplicity and to make a more convenient and comply with the editor’s prior suggestion, and to avoid back and forth, we decided and* ***removed*** *the figure from the manuscript.*

5. If the reviewer comments include a recommendation to cite specific previously published works, please review and evaluate these publications to determine whether they are relevant and should be cited. There is no requirement to cite these works unless the editor has indicated otherwise.

**Response:**

*Thank you, and noted.*

**Response to reviewer comments.**

**Reviewer #1**

1. Quantitative results: The abstract reports the prevalence of virological failure as 14.9% and the incidence density as 27.2 per 10,000 person-months. It would be helpful also to report the confidence intervals for these estimates to provide a sense of their precision.

**Response:**

*We thank the reviewer for this suggestion. We have added the 95%CI and read as “14.9%, (95% CI 11, 19), lines 31 to 32, lines 324 and 340 of the revised clean version of the manuscript.*

2. Qualitative results: The methods section provides a good overview of the quantitative methodology, but has less detail on the qualitative methodology. The authors should provide more information on the following:

a. Sampling: How were the participants for the KIIs and FGDs selected? What were the specific inclusion and exclusion criteria?

**Response:**

*We thank the reviewer for this suggestion. “Both KII and FGD participants were selected purposefully. The KII participants were selected based on their status as ART service users, and the FGD participants were selected based on their specific professional roles as HIV service providers in the hospitals and program managers.*

*Adult PLHIV aged 18 years and older, who were currently taking second-line ART, had a regular follow-up visit to the hospitals during the data collection period, and willingly provided their informed consent for the interview, have been included in KII. Individuals who were on first-line ART, under 18 years old, or who did not volunteer were excluded from the KII”.*

*Moreover, HIV service providers (including ART clinicians, pharmacists, case managers, data clerks, and adherence supporters at selected hospitals) and HIV program managers at the regional and sub-city health bureau level were included in the FGD. Individuals who did not hold one of these specific roles were excluded from the FGDs. Lines 142 to 153 of the revised version of the manuscript.*

b. Data Collection: How were the KIIs and FGDs conducted? What were the key topics covered in the interview and discussion guides?

**Response:**

*To ensure consistency, KII and FGD guiding questions were developed, and all data collectors were oriented on these questions before data collection commenced, ensuring the information was gathered accordingly.*

*The key topics covered during the interviews and discussions centered on challenges, developing effective and targeted strategies to improve ART adherence and achieve optimal health outcomes for PLHIV taking second-line ART. Lines 213 to 218 of the revised version of the manuscript.*

c. Data Analysis: How was the qualitative data analyzed? What specific thematic analysis approach was used?

**Response:**

*The qualitative data analysis was conducted using a thematic analysis approach, by Atlas.ti software, Version 24. The qualitative data from the KIIs and FGDs were imported into Atlas.ti software, where a systematic thematic analysis was conducted by grouping the documents, generating initial codes, refining and grouping the coded segments, and then performing final analysis and visualization. Hence, the themes were generated directly from the data without trying to fit them into pre-existing theory or framework. The specific theme the researcher used was the inductive thematic analysis. Lines 238 to 244 of the revised version of the manuscript.*

d. Mixed-Methods Integration: The methods section mentions that the quantitative and qualitative data were integrated through triangulation, but it does not provide any detail on how this was done. The authors should describe the specific steps they took to integrate the two datasets.

**Response:**

*We thank the reviewer for this suggestion. We used the following general steps: Independent analysis, conversion, and comparison, point-by-point triangulation, and integrated interpretation.*

*The quantitative and qualitative data were analyzed separately using SPSS software version 28 and Atlas ti software version 24, respectively. Key findings from both datasets were converted into a common format. Quantitative results were presented as statistical outcomes, while qualitative themes were converted into descriptive statements. The results were systematically compared on a point-by-point basis to identify areas of convergence, divergence, and expansion. The final discussion and conclusion synthesized these compared findings. Lines 248 to 254. Moreover, it was illustrated in S file 1 (line 116), in the revised version of the manuscript.*

3. The quantitative results are generally well-presented, but the tables and figures could be improved. For example, Table 2 is very dense and difficult to read. The authors should consider splitting this table.

**Response:**

*We thank the reviewer for the thoughtful suggestion. We fully appreciate the importance of clarity and readability, especially when presenting dense quantitative data. In this case, however, we intentionally structured the table as a single, continuous unit to preserve the integrity and coherence of the dataset. Splitting it into separate tables may risk fragmenting the narrative and obscuring key relationships across variables. We have carefully reviewed its layout and ensured that it remains accessible while conveying the full message to readers who seek comprehensive insight. With due respect to the reviewer’s suggestion, we believe maintaining it as one unified table could best serve the analytical purpose of the manuscript.*

4. Presentation of Qualitative Results: The qualitative results are presented as a series of quotes, which is effective in illustrating the key themes. However, the authors should provide more of their own analysis and interpretation of the quotes, rather than simply presenting them.

**Response:**

*We thank the suggestion. We added some analysis on the selected quotes, as indicated in the revised version of the manuscript.*

5. Integration of Quantitative and Qualitative Results: The results section presents the quantitative and qualitative results separately. The authors should consider integrating the two datasets more closely, for example, by using quotes to illustrate the quantitative findings.

**Response:**

*We appreciate the thoughtful suggestion. In our study, we employed both quantitative (survey-based) and qualitative (KII and FGD) methodologies, each designed to address distinct research questions. The survey data specifically explored the following areas:*

- *Reasons for switching to second-line ART regimens*
- *opportunistic infections among individuals on second-line ART*
- *Time to OI in PLHIV receiving second-line ART*
- *Magnitude and associated risk factors for virological failure (VF) in second-line ART*
- *Time to treatment failure among patients on second-line ART*

*To enhance clarity and alignment, we have re-labeled the qualitative: KII and FGD results to reflect the two additional thematic areas under investigation:*

- *Reasons for virological failure derived from key informant interviews*
- *Factors influencing virological failure in second-line ART based on focus group discussions*

*Final interpretation was achieved through a synthesis of both data streams, enabling mutual validation and deeper contextual understanding. For example, the survey revealed a statistically significant association between non-disclosure of HIV status and increased risk of virological failure. This quantitative finding was strongly reinforced by focus group participants, including hospital-level ART providers and program managers, who emphasized that non-disclosure often limits access to social support, thereby undermining medication adherence and negatively affecting treatment outcomes.*

6. Discussion: The discussion section provides a good overview of the key findings, but it could be strengthened by more fully integrating the quantitative and qualitative findings. The authors should discuss how the qualitative findings help to explain the quantitative results.

**Response:**

*We appreciate the suggestion, and the recent discussion of results was mainly derived from the triangulation of qualitative and quantitative datasets. This is addressed, as indicated (for sample) in lines 638-641, 647,667, 672-673, 679, and 683-687 in the revised version of the manuscript.*

7. Limitations: The authors acknowledge some of the limitations of the study, but they could be more comprehensive. For example, they should discuss the potential for selection bias in the qualitative component of the study.

**Response:**

*Thank you for highlighting this important point. We fully recognize the potential for selection bias in qualitative research and appreciate your suggestion to address it more explicitly. In response, we have made revisions to the Methods section of the revised manuscript to reflect this concern. Specifically, we now clarify that participant selection was guided by a clearly defined inclusion criterion, applied consistently by a trained data collector under close supervision to minimize bias. We have added the following statement to the Methods section (lines 223–225) of the revised manuscript: “To mitigate potential selection bias, participants were recruited based on a pre-tested standardized inclusion criterion by a trained research data collector, with oversight from the study supervisor to ensure procedural fidelity.”*

**Reviewer #2:**

Thank you for submitting your interesting work. Unfortunately, as reviewer, I did not find the manuscript coehsive but instead appeared to be an attempt to include all collected data regardless of relevance to the scientific questions at hand.

This paper needs major revisions with an eye towards the following:
1) Determine the most important scientific question regarding 2nd-line ART and limit your analysis to answering that question even if it means foregoing presentation of data that I am sure you have painstakingly collected

**Response:**

*We thank the reviewer for this insightful and constructive recommendation. We fully acknowledge the importance of focusing the manuscript on a clearly defined and impactful scientific question. Accordingly, we have refined the scope of our analysis to emphasize the magnitude of virological failure and its most significant risk factors among people living with HIV receiving second-line ART in Addis Ababa, Ethiopia. This central question is both timely and critical for informing targeted public health interventions, optimizing resource allocation for second- and third-line ART, and guiding clinical decision-making for high-risk populations.*

*While we recognize the value of brevity in scientific reporting, we believe that the inclusion of both quantitative and qualitative data analyzed concurrently through a mixed cohort design (lines 109–110) enhances the depth and applicability of our findings. In line with this, our research result seems bulk, but as clarified in the revised manuscript (lines 101–106), this approach allows for a more nuanced understanding of the contextual factors influencing treatment outcomes, which we consider essential for policymakers, program managers, and researchers working to strengthen ART programs.*

*We have carefully reviewed and streamlined the presentation of results to maintain clarity and relevance, while preserving the integrity of the evidence base. We trust that this focused revision aligns with the reviewer’s guidance and strengthens the manuscript’s scientific contribution.*

2)When presenting your results avoid the use of subjective statements justifying the presentation of your results. Your results should speak for themselves and if elaboration is needed, reserve for the discussion

**Response:**

*We thank the suggestion, and to the best of our capacity, we revised the result to be more objective by avoiding the subjective nature of the result findings*

3) Your introduction should be limited to the key scientific question with background followed by a brief statement justifying your approach to answering the question

**Response:**

*We thank the suggestion, we refined it accordingly*

4) Limit your figures to 2-3 to amplify your results and justify your conclusions
Also would like to see you show data on prior regimens at the time of switch. Given time period you are reporting on, a reader would need to know if the prior regimen was NNRTI-based or DTG-based. If NNRTI-based, I am not sure this would be very relevant to the DTG-era but you could try to make a case that it is

**Response:**

*We sincerely appreciate the reviewer’s thoughtful feedback and constructive suggestions.*

*We tried to minimize the number of figures to 4 to present our results based on the stated research question and the proposed objective to streamline the narrative while preserving the integrity of the evidence base. We have moved some of the figures to S files, and now this matches the number of figures allowed by the journal.*

*Concerning prior ART regimens, we sincerely appreciate the reviewer’s thoughtful observation regarding the importance of prior ART regimens at the time of switch. We fully agree that, given the evolving treatment landscape and the transition to DTG-based regimens, such data can offer valuable context, particularly in interpreting virological outcomes.*

*In our study, participants were indeed stratified by prior regimen (NNRTI-based vs. DTG-based), and we had the raw data reflecting these histories. However, in the interest of maintaining clarity and focus, the manuscript primarily concentrated on second-line ART outcomes, not included in the recent manuscript. This decision was made to avoid excessive detail that might detract from the central narrative.*
