# Supplementary material for: Virological failure and risk factors among people living with HIV taking second-line ART in Addis Ababa, Ethiopia
Source: PLoS One. 2026 Feb 2;21(2):e0330581. doi: 10.1371/journal.pone.0330581 (PMC12863470; doi:10.1371/journal.pone.0330581)
Supplement: Attachment — Submitted filename: Rebuttal letter 4 R1.docxBB Feyissa.docx [file pone.0330581.s006.docx]

9 November 2025

The Academic Editor, PLOS ONE

**SUBMISSION OF Re-REVISED MANUSCRIPT:** PONE-D-25-41462R1

Attached is the re-revised version of our manuscript entitled “Virological failure and risk factors among people living with HIV taking second-line ART in Addis Ababa, Ethiopia**”.** We thank you once again for giving us improve the manuscript. All comments and suggestions by the editor and reviewers have been addressed as outlined within this document in a point-by-point fashion, and all changes to the manuscript are indicated with “track changes”.

Please don't hesitate to contact us if you have any questions. All correspondence regarding the manuscript should be addressed to Bekelech Bayou, at [*bekelechbayou@gmail.com*](mailto:bekelechbayou@gmail.com)*.*

Sincerely,

Bekelech B. Feyissa

*Addis Ababa,*

*Ethiopia*

**Intro:**

*We would like to sincerely thank once again the editor and the reviewers for the time taken to re-review our work and provide constructive feedback. Your comments have been instrumental in improving the quality of the manuscript. Below, we detail how we have addressed each point as raised by the editor and reviewers*.

**Editor’s suggestion**

- The reviewers agree attempts have been made to revise the manuscript. However, they have additional comments and suggestions for improvement. Kindly pay attention to the issues about mixed methods raised by the second reviewer in previous submission. This aspect still need clarification.

**Author Response:**

*Thank you very much for sharing the issues and the positive suggestions. We appreciate the reviewers’ continued engagement and the editor’s guidance. To clarify our mixed methods approach, we conducted independent quantitative and qualitative analyses using SPSS v28 and Atlas.ti v24, respectively. Key findings from quantitative and qualitative were themed point-by-point to identify convergence, divergence, and expansion, culminating in an integrated interpretation (lines 245–256; S file 1).*

*The revised manuscript emphasizes how this concurrent mixed cohort design enhances contextual understanding of research findings. We streamlined the presentation for clarity while preserving analytical depth.*

*Quantitative data addressed treatment switching, opportunistic infections, and virological failure. Qualitative data (KII and FGD) were re-labeled to reflect thematic alignment, focusing on reasons and factors influencing virological failure (lines 383 and 498). For instance, the statistical link between HIV non-disclosure and virological failure was validated by qualitative insights from ART providers and program managers, highlighting its impact on adherence and social support.*

*Moreover, the recent discussion of results was mainly derived from the triangulation of qualitative and quantitative datasets. This is addressed, as indicated (for sample) in lines 640, 646,667-672, 673-678, and 682-686 in the revised version of the manuscript.*

*We trust these revisions address the concerns raised and strengthen the manuscript’s methodological clarity and scientific contribution*

Reviewers' comments:

**Reviewer #1:** Minor comments to be addressed:
1. In the 'Strengths and limitations of the study' section, authors should check for duplications. Review lines 734-761 of the clean copy and address redundant text.

Author Response:

*Thank you very much for raising the points. We thought the reviewer used the tracked change version of the manuscript (Lines 734-761). However, it lies b/n lines 691-703 in the Clean Version of the previously submitted version.*

*We conducted a thorough review, avoiding redundant content and making necessary revisions as indicated in lines 689- 700 of the recent revised version of the manuscript.*

2. Check inconsistencies in figure numbering. For example figures 24A and B and figure 35 seem to be out of sequence.

Response:

*Thank you for highlighting this. The apparent discrepancy may have arisen from referencing the tracked-changes version of the manuscript. For your reference, what was previously labeled as Figure 4A was revised to Figure 2A, and Figure 5 was renumbered as Figure 3 in the previously submitted version. These adjustments were accurately reflected in the clean version of the manuscript, which was submitted to clearly present all revisions made in response to reviewer and editor feedback.*

*Nonetheless, we have carefully reviewed the figure numbering again and confirm that all figures are now correctly labeled and presented in the appropriate sequence.*

3. While the explanation for not splitting table 2 is accepted, it is still challenging in terms of readability . Improving its layout may be helpful.

Response:

*Thank you for understanding our concern and allowing us to maintain it as it is, and we did an improvement on the layout of the table as much as possible to increase more e readability.*

4. A final proofread will be useful to check consistency in the terminology. For example 'second line' vs '2nd line'

Authors Response:

*Thank you for Thank you for the helpful suggestion. The senior authors have conducted a final round of proofreading to ensure grammatical accuracy and consistency in terminology throughout the manuscript. As part of this process, instances of “2nd line” were revised to “second-line” to maintain uniformity in expression.*

**Reviewer #2:** (No Response)

Authors Response:

*No specific comments*

*…………………………………………,,,,,,,,,,……………………………………….*
